# Editorial: Microbial nanotechnology: a new frontier in microbiology

**DOI:** 10.3389/fmicb.2023.1296573

**Published:** 2023-10-19

**Authors:** Madan L. Verma, Paul J. Joseph, Didem Sen Karaman

**Affiliations:** ^1^Department of Biotechnology, School of Basic Sciences, Indian Institute of Information Technology Una, Himachal Pradesh, India; ^2^Institute for Electronics and Nanotechnology, Georgia Institute of Technology, Atlanta, GA, United States; ^3^Department of Biomedical Engineering Cigli, Faculty of Engineering and Architecture, Izmir Katip Celebi University, Izmir, Türkiye

**Keywords:** microbial nanotechnology, biogenic material, production, application, environmental sustainability

Microbial nanotechnology, microorganisms-driven nanobiotechnology, is an emerging area of microbiotechnology that has leveraged biotechnological processes. Bioprospecting of microorganisms enables the production of a plethora of diverse nanoscale materials such as organic nanomaterials, metals, and their oxide nanomaterials, etc. (Verma et al., 2022). Microbial nano-factories route utilizes a green facile approach for the production of biogenic nanomaterials as compared to the alternative synthetic routes such as chemical, physical, and physico-chemical approaches. Microbial nanomaterials are endowed with the functionalized bioactive groups that render the improvement in the stabilities as well as functionality at the nanoscale level. These microbial nanoproducts have been primarily employed as the robust carriers for intact delivery/utilization of bioactive components to the tailor made applications ranging from agri-food to the drug industries (Chamundeeswari et al., 2019). Microbial nanomaterials have been employed for the decontaminating of the environmental toxic materials by biocatalytically degrading harmful pollutants emanated from the industrial effluents to the harmless byproducts (Verma, 2017; Verma et al., 2020). Thus, microbial nanobiotechnology possesses a broad scope of application constituting a cost-efficient methodology in microbial nanomanufacturing and may offer massive profits to society in the near future.

Impact of heavy metals and pathogenic bacteria can be minimized for the sustainable aquaculture industry with the advent of green nanotechnology. In this regard, Saad et al. developed the efficient methodology for the production of selenium nanoparticles of 77 nm size using the bacterium *Bacillus subtilis* AS12. Through bacterial mediated biosynthesis of selenium nanoparticles, nanoparticle stability in terms of shapes and sizes were provided by enriched bacterial suspension of functional bioactive components namely flavonoids, and secondary metabolites. These nanoparticles were tested against the accumulation of two heavy metals (Cd and Hg) and a pathogenic bacterial load of *Aeromonas hydrophila* in the Nile tilapia fish, *Oreochromis niloticus*. Further authors recommended that the biogenic selenium nanoparticles may be ideal for use in the polluted water to minimize the side effects of pathogenic microbes and heavy metals; and thus, enhancing the productivity of the aquaculture industry.

Fungal infection to the agricultural crops can lead to the severe loss to the agriculture productivity. Recently, the agricultural industry can be protected against the fungal infection with the aid of green nanotechnology (Narware et al.). Narware et al. developed the facile methodology for the production of silver nanoparticles of size 48 nm with the aid of *Trichoderma harzianum* culture filtrate. The culture filtrate was enriched with bioactive molecules for providing the capping agent for the biosynthesis of robust silver nanoparticles. Silver nanoparticle validity was done using the advanced microscopy and spectroscopy techniques such as UV-Vis, FTIR, EDX, XRD, DLS, AFM, and SEM. Further, the given nanoparticles gave better anti-fungal results for *in-vitro* and *in-vivo* treatments to inhibit the pathogenic fungus namely *Alternaria solani* responsible for the late blight disease. The authors have demonstrated the anti-fungal effects of green silver nanoparticles more than the standard fungicide. Thus, this leads to an encouraging outcome in the near future to explore the fully biosynthesized green nanoparticles over the chemically synthesized fungicide.

Researcher (Altammar) discussed the critical survey for the production of metallic nanoparticles and their associated challenges in agri-food industries, medicine industry, defense industry, environment, electronics industry, and automotive industry. Ogunyemi et al. employed an innovative approach for developing the biogenic nanoparticles. Magnesium oxide (MgO) nanoparticles and manganese oxide (MnO_2_) nanoparticles and of sizes 12.5 and 9.8 nm, were biosynthesised using the lysate of *Xanthomonas* oryzae pv. *oryzae* (Xoo) bacteriophage X_3_ with the target application to plant protection from the bacterial pathogenic. Researchers investigated the minimize the ill-effect of bacterial leaf blight disease that causes yield loss of rice plants, the staple food of the region. The physicochemical characterization of bacteriophage mediated nanomaterials were characterized using sophisticated spectroscopy and microscopy methods. The authors have employed the chlorophyll fluorescence to determine the safety/toxicity of these nanoparticles to the plants. Thus, authors have successfully demonstrated the effective alternative biogenic production of MgO and MnO_2_ nanoparticles to circumvent the plant bacterial disease without any phytotoxic effect.

Murali et al. reviewed the microorganism-assisted synthesis of zinc oxide nanoparticles (ZnO NP). Researchers discussed the detailed production strategies with the potential applications of bioactive molecules. Further toxicological effects of zinc oxide nanoparticles on the environmental ecosystem were discussed. Furthermore, the potential applications of ZnO NP in different sections such as anti-bacterial, anti-fungal, anti-cancer, antioxidant activity, photocatalytic activity, drug delivery system, wound healing. The authors have discussed the production of intracellular and extracellular methods of nanoparticles production. The researchers have also highlighted the limitations as well as exact role of reproducibility of ZnO NP synthesis due to unavailability of stable microbes that further requires a rigorous screening method for the selection of robust microbes.

Microbial nanotechnology is still in the infancy stage that has provided the vast opportunities for further explorations and innovations. The present Research topic demonstrated the impacts of microorganism driven nanotechnology for improving the processes of agri-food industries. And further microbial technology provides the green technologies for sustainable environment by detoxification and neutralization of the toxic waste materials. As per the above discussed research, it is clearly demonstrated that the role of green/microbial nanotechnology is expanding in diverse areas ranging from food to the environment. The results found at the laboratory scale is quite interesting. However, the massive scale production of these nanoparticles is the need of the hour. Thus, it may be concluded with the recently completed studies, that microbial nanotechnology will be a boon for the sustainable commodity production in near future.

## Author contributions

MV: Conceptualization, Writing—original draft, Writing—review and editing. PJ: Writing—review and editing. DS: Writing—review and editing.
